# Sex-specific effects of central adiposity and inflammatory markers on limbic microstructure

**DOI:** 10.1016/j.neuroimage.2019.02.007

**Published:** 2019-04-01

**Authors:** Claudia Metzler-Baddeley, Jilu P. Mole, Erika Leonaviciute, Rebecca Sims, Emma J. Kidd, Benyamin Ertefai, Aurora Kelso-Mitchell, Florence Gidney, Fabrizio Fasano, John Evans, Derek K. Jones, Roland J. Baddeley

**Affiliations:** a Cardiff University Brain Research Imaging Centre (CUBRIC), School of Psychology, Cardiff University, Maindy Road, Cathays, Cardiff, CF24 4HQ, UK; b Psychological Medicine and Clinical Neurosciences, School of Medicine, Cardiff University, Maindy Road, Cathays, Cardiff, CF24 4HQ, UK; c School of Pharmacy and Pharmaceutical Sciences, Cardiff University, Redwood Building, King Edward VII Avenue, Cardiff, CF10 3NB, UK; d Siemens Healthcare, Head Office, Sir William Siemens Square, Surrey, GU16 8QD, UK; e School of Psychology, Faculty of Health Sciences, Australian Catholic University, Melbourne, Victoria 3065, Australia; f Experimental Psychology, University of Bristol, 12a Priory Road, BS8 1TU, UK

## Abstract

Midlife obesity is a risk factor of late onset Alzheimer's disease (LOAD) but why this is the case remains unknown. As systemic inflammation is involved in both conditions, obesity-related neuroinflammation may contribute to damage in limbic structures important in LOAD. Here, we investigated the hypothesis that systemic inflammation would mediate central obesity related effects on limbic tissue microstructure in 166 asymptomatic individuals (38–71 years old). We employed MRI indices sensitive to myelin and neuroinflammation [macromolecular proton fraction (MPF) and *k*_*f*_] from quantitative magnetization transfer (qMT) together with indices from neurite orientation dispersion and density imaging (NODDI) to investigate the effects of central adiposity on the fornix, parahippocampal cingulum, uncinate fasciculus (compared with whole brain white matter and corticospinal tract) and the hippocampus. Central obesity was assessed with the Waist Hip Ratio (WHR) and abdominal visceral and subcutaneous fat area fractions (VFF, SFF), and systemic inflammation with blood plasma concentrations of leptin, adiponectin, C-reactive protein and interleukin 8. Men were significantly more centrally obese and had higher VFF than women. Individual differences in WHR and in VFF were negatively correlated with differences in fornix MPF and *k*_*f*_, but not with any differences in neurite microstructure. In women, age mediated the effects of VFF on fornix MPF and *k*_*f*_, whilst in men differences in the leptin and adiponectin ratio fully mediated the effect of WHR on fornix MPF. These results suggest that visceral fat related systemic inflammation may damage myelin-related properties of the fornix, a key limbic structure known to be involved in LOAD.

## Highlights

• Central adiposity is linked to apparent myelin/inflammatory damage in the fornix.
• Central adiposity is not linked to differences in apparent neurite microstructure.
• Men were more centrally obese and had higher visceral fat fractions than women.
• In women, age mediated the correlation between visceral fat and fornix myelin.
• In men, adipokines mediated the correlation between WHR and fornix myelin.

## 1 Introduction

Obesity is globally on the rise (World Health Organisation, 2018; Alzheimer's Research UK, 2014) and has become an epidemic in many Western countries. In the UK, two-thirds of adults are overweight or obese, defined by a Body Mass Index (BMI) of >25 kg/m^2^ or 30 kg/m^2^ respectively. Western-style diet and sedentary lifestyles contribute to obesity risk and its related diseases including metabolic syndrome, type 2 diabetes, and cardiovascular disease (Cox et al., 2015). Several epidemiological studies have also identified a positive association between midlife obesity and the incidence of late onset Alzheimer's disease (LOAD) (estimated risk ratio of ∼1.4) (Beydoun et al., 2008; Pedditizi et al., 2016). While the effects of excessive adiposity are complex and involve multiple immune, metabolic, and endocrine factors, it is increasingly recognised that persistent, low-grade inflammation may play a key role in obesity and its related diseases (Cox et al., 2015; Valcarcel-Ares et al., 2018). Similarly, microglia-mediated immune responses are thought to play an important role in LOAD development (Dansokho and Heneka, 2018; Heneka et al., 2015; Sarlus and Heneka, 2017; Tejera and Heneka, 2016), and it has therefore been proposed that obesity induced gut dysbiosis may trigger microglia mediated neuroinflammation, and that this in turn may contribute to disease development (Sochocka et al., 2018; Venegas et al., 2017), potentially many years prior to the onset of LOAD (Jack et al., 2013). If that was the case, one may expect adverse effects of obesity-related neuroinflammation to manifest in brain regions involved in LOAD in asymptomatic individuals.

Here, we investigated whether systemic inflammation would mediate the relationship between central obesity and brain microstructure in 166 asymptomatic individuals from the Cardiff Ageing and Risk of Dementia Study (CARDS) (38–71 years old) (Table 1). Going beyond previously adopted diffusion tensor imaging (DTI) analyses of obesity-related microstructural brain differences (Alfaro et al., 2018; Kullmann et al., 2015), we employed multi-compartment diffusion imaging indices of neurite microstructure from neurite orientation dispersion and density imaging (NODDI) (Zhang et al., 2012) and indices from quantitative magnetization transfer (qMT) (Sled, 2017) that provide better sensitivity to myelin and inflammation in white matter than DTI (Ceckler et al., 1992; Koenig, 1991; Levesque et al., 2010; Schmierer et al., 2007; Serres et al., 2009a).

**Table 1:** Summary of demographic, cognitive, genetic and lifestyle-related health information for men and women.

Mean (SD)	Men (n = 72)	Women (n = 94)	Statistic (p-value)
Age (in years)	56 (8.3)	55.6 (8.2)	*ns*
Years of education	16.6 (3.4)	16.5 (3.3)	*ns*
NART	116.9 (6.4)	116.6 (6.9)	*ns*
MMSE	29 (1.0)	29.2 (0.9)	*ns*
Positive Family History dementia	36.1	35.1%	*ns*
*APOE* Genotype	44.4% ε4+, 55.6% ε4-	34.4% ε4+, 65.6% ε4-	*ns*
Central obesity (Waist Hip Ratio)	84.7%	44.7%	χ^2^(1) = 28.34 (<0.0001)
Overweight/Obese (BMI)	50%/18%	39.4%/21.3%	*ns*
Subcutaneous fat area fraction	0.35 (0.1)	0.41 (0.1)	t(128) = 3.5 (0.001)
Visceral fat area fraction	0.22 (0.06)	0.19 (0.06)	t(128) = 3.8 (0.002)
Systolic Hypertension	36.1%	22.3%	*ns*
Smokers	4.2%	6.4%	*ns*
Diabetes	2.8%	1.1%	*ns*
Statins	13.9%	3.3%	χ^2^(1) = 6.5 (0.01)
Alcohol units per week	11.5 (11.9)	4.3 (5.0)	t(156) = 5.2 (<0.0001)
Physical activities (Median hours per week)	11.9 (12.7)	9.2 (11.5)	*ns*
Adiponectin log10 ng/ml	3.9 (0.26)	4.1 (0.22)	t(146) = 4.64 (<0.0001)
Leptin log10 pg/ml	3.7 (0.39)	4.3 (0.37)	t(146) = 9.02 (<0.0001)
C-Reactive Protein log10 ng/ml	3.0 (0.45)	3.1 (0.49)	*ns*
Interleukin-8 log10 pg/ml	0.7 (0.14)	0.7 (0.16)	*ns*

Abbreviations: *APOE* = Apolipoprotein E, BMI = Body Mass Index, MMSE = Mini Mental State Examination, NART = National Adult Reading Test.

**Fig. 1:**
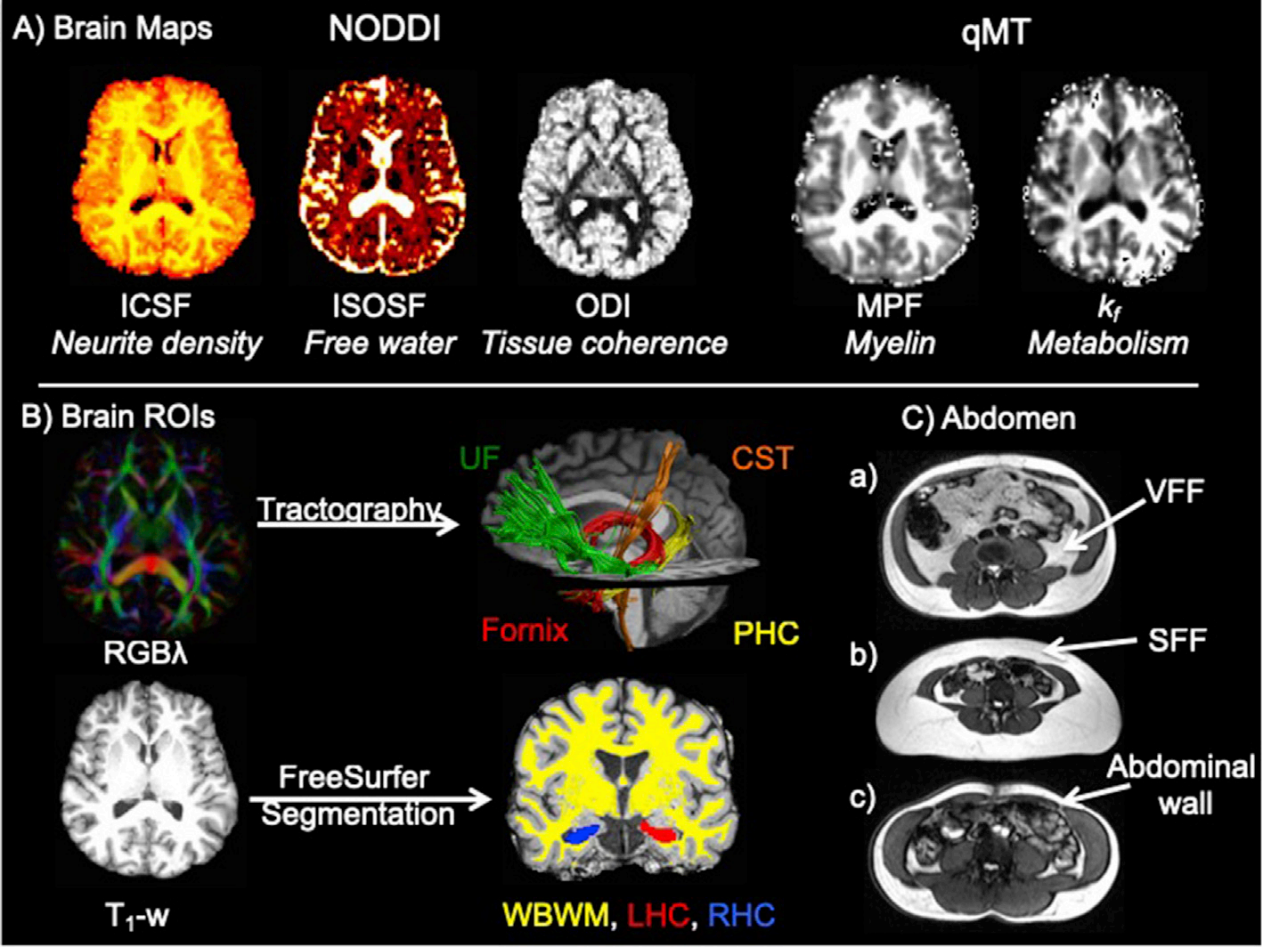
A) displays the MRI modalities and maps from dual-shell high angular resolution imaging (HARDI) and quantitative magnetization transfer (qMT) imaging. HARDI data were modelled with neurite orientation dispersion and density (NODDI) yielding maps of intracellular signal fraction (ICSF), isotropic signal fraction (ISOSF) and orientation density index (ODI). qMT based maps were the macromolecular proton fraction (MPF) and the forward exchange rate *k*_f_. B) Mean indices of the metrics were extracted from the left hippocampus (LHC) (red), right hippocampus (RHC) (blue), whole brain white matter (WBWM) mask (yellow), fornix (red), parahippocampal cinguli (PHC) (yellow), uncinate fasciculi (UF) (green) and corticospinal tract (CST) (orange). Hippocampi and WBWM were segmented from T_1_-weighted images with FreeSurfer version 5.3 and fornix, PHC, UF and CST were reconstructed with damped-Richardson Lucy spherical deconvolution (dRL) based deterministic tractography on colour coded principal direction maps (RGBλ). C) Examples of abdominal images from a) a 51 years old male with larger visceral (VFF) than subcutaneous fat area fraction (SFF), b) a 52 years old female with larger SFF than VFF and c) a 60 years old female with low SFF and VFF. All abdominal images were acquired with the same field of view of 480 × 390 mm.

The qMT technique exploits the process of magnetization transfer (MT) between free and macromolecular bound protons. In white matter, MT is dominated by myelin (Ceckler et al., 1992; Koenig, 1991), and is also sensitive to neuroinflammation (Harrison et al., 2015; Levesque et al., 2010; Schmierer et al., 2007; Serres et al., 2009a). The macromolecular proton fraction (MPF), provides an index of apparent myelin content of white matter (Fig. 1A, Table 2). In addition, the rate of the MT process *k*_*f*_ was found to be sensitive to acute neuroinflammation in response to typhoid vaccination in the insular cortex, an area which also exhibited vaccination-induced increases in fluorodeoxyglucose positron emission tomography metabolism that correlated with inflammation-induce fatigue (Harrison et al., 2015). Similarly, *k*_*f*_ has been proposed to reflect metabolic efficiency of mitochondrial function (Giulietti et al., 2012) that may contribute to inflammatory mechanisms (Fig. 1A, Table 2).

**Table 2:** Overview of the quantitative microstructural indices and their interpretation in white matter.

MRI modality	Index	Apparent white matter property	Hypothesised changes in central obesity
Diffusion NODDI	ICSF	Axon density	Reduction
	ODI	Axon dispersion	Increase
	ISOSF	Free water	Increase
qMT	MPF	Myelin Neuroinflammation	Reduction
	*k*_*f*_	Neuroinflammation Mitochondrial metabolism	Increase in acute inflammation (Harrison et al., 2015); Reduction in low-level inflammation (Johnson et al., 2012)

Abbreviations: ICSF = intracellular signal fraction, ISOSF = isotropic signal fraction, *k*_*f*_ = forward exchange rate, MPF = macromolecular proton fraction, NODDI = neurite orientation dispersion and density imaging, ODI = orientation dispersion index, qMT = quantitative magnetization transfer.

These qMT measurements were contrasted with MRI estimates of neurite microstructure from NODDI (Zhang et al., 2012) yielding separate indices of apparent neurite density [intra-cellular signal fraction (ICSF)], neurite orientation dispersion (ODI), and free water [isotropic signal fraction (ISOSF)] (Fig. 1A) (Table 2). We propose that the combination of qMT and NODDI indices will allow us to dissociate any obesity-related effects on myelin/inflammation/metabolism from neurite density/orientation components in white and grey matter MTL regions.

As midlife obesity is a risk factor of LOAD, and LOAD pathology is proposed to spread from the hippocampal formation *via* limbic white matter pathways such as the fornix (Plowey and Ziskin, 2016) to the neocortex (Braak and Del Trecidi, 2015), we hypothesised that obesity-related differences would disproportionally affect limbic white and grey matter regions, i.e. the fornix, parahippocampal cinguli, uncinate fasciculi, and the hippocampi, relative to whole brain and cortico-spinal-motor white matter (Kullmann et al., 2015; Metzler-Baddeley et al., 2013). To test this hypothesis, mean values of qMT and NODDI indices were extracted from all regions (Fig. 1B). Principal component analysis (PCA) was then used i) to assess the relationships between the different microstructural indices in white and grey matter and ii) to reduce the dimensionality of the data for further correlational analyses of obesity-brain relationships.

Most previous imaging studies into obesity have investigated differences in BMI, an index that does not capture variation in body fat distributions (Adab et al., 2018). There is increasing recognition that it is not body fat *per se* but visceral rather than subcutaneous fat which leads to adverse health effects and increased risk of metabolic syndrome and mortality (Koster et al., 2010, 2015). We therefore assessed obesity not only with BMI, but also with metrics of central adiposity, notably the Waist Hip Ratio (WHR) and MRI indices of visceral fat area fractions (VFF) and subcutaneous fat area fractions (SFF) (Fig. 1C). The examples in Fig. 1C demonstrate the considerable individual variation in the distribution of abdominal subcutaneous and visceral fat.

Individual differences in systemic inflammation were measured with plasma concentrations of high sensitivity C-Reactive Protein (CRP) and interleukin-8 (IL-8) (Swardfager et al., 2010), as well as with leptin and adiponectin, two adipokines that are involved in glucose control and the modulation of inflammatory responses (Arnoldussen et al., 2014; Doherty, 2011; Flak and Myers, 2016; Myers et al., 2008; Ryan et al., 2003). Leptin is known to up-regulate pro-inflammatory cytokines, while adiponectin has anti-inflammatory properties and down-regulates the release and the expression of pro-inflammatory cytokines (Lopez-Jaramillo et al., 2014). Central obesity is associated with an imbalance between increased leptin and reduced adiponectin, and hence the ratio between leptin and adiponectin is a sensitive marker of visceral fat related inflammatory states (Lopez-Jaramillo et al., 2014).

To disentangle effects of obesity-related inflammation from other demographic and health-related variables, and to study the potential link with risk factors of LOAD, information about education, hypertension, alcohol consumption, physical activity, *APOE* genotype and family history of dementia were also collected and included in the analysis (Dommermuth and Ewing, 2018; Ricci et al., 2017) (Table 1). As men and women are known to differ in their body fat composition (Bredella, 2017), we also explored any potential sex-related differences in adiposity indices, demographic, health, and genetic variables (Table 1).

Inter-individual differences in central obesity, specifically in abdominal visceral fat, were hypothesised to be associated with differences in MRI markers of apparent myelin, inflammation, and metabolism (MPF and *k*_*f*_) (Table 2) in limbic white matter pathways, notably the fornix (Yu et al., 2017). We also expected obesity to be accompanied by increases in ISOSF reflecting free water contamination potentially due to unspecific tissue loss (Table 2). In addition, based on previous DTI findings (Kullmann et al., 2015) one might expect obesity to reduce the density of axons (ICSF) or alter their orientation dispersion in white matter (Table 2). The application of the microstructural metrics to the hippocampus should be seen as exploratory, as microstructural indices are more difficult to interpret in grey than in white matter due to the former's more complex organisation. For this reason, and to test our assumption that qMT and NODDI indices would provide separable measurements of uncorrelated tissue properties, we explored their dimensionality in white and grey matter regions separately using PCA.

## 2 Methods

### 2.1 Participants

Over a period of three years (2014–2017), n = 211 community-dwelling individuals between 35 and 75 years of age were recruited for CARDS from local Cardiff University databases and *via* internet and poster advertisements. The study was approved by the School of Psychology Research Ethics Committee at Cardiff University (EC.14.09.09.3843R2) and all participants gave written informed consent in accordance with the Declaration of Helsinki. Exclusion criteria were a history of neurological disease (e.g. Multiple Sclerosis, Parkinson's disease, Huntington's disease), psychiatric disease [e.g. schizophrenia, bipolar disorder, depression requiring hospitalization or a current score of >15 in the Patient Health Questionnaire (PHQ-9) indicating severe depression], moderate to severe head injury with loss of consciousness, drug or alcohol dependency, high risk cardio-embolic source (mitral or severe aortic stenosis, severe heart failure, cardiac aneurysm), known significant large-vessel disease (i.e. more than 50% stenosis of carotid or vertebral artery, known peripheral vascular disease, coronary bypass or angioplasty) and MRI contraindications (e.g. pacemaker, cochlear implants, metal pins, stents, screws etc.). Demographic and health information including information about genetic and lifestyle risk factors of dementia was collected for all 211 volunteers. Here we report data from n = 166 participants who also underwent MRI scanning at CUBRIC. Table 1 provides a summary of the demographic, health and genetic information for these 166 participants, separately for men (n = 72) and women (n = 94).

### 2.2 Assessment of body composition/adiposity

Abdominal adiposity was assessed by measuring participants' waist and hip circumferences to calculate WHR following the World Health Organisation's recommended protocol (World Health Organisation, 2008). Abdominal obesity was defined as a WHR ≥0.9 for males and ≥0.85 for females. BMI was calculated from participants' height and weight. Normal weight was defined as a BMI of 18–24.9 kg/m^2^, overweight as BMI of 25–29.9 kg/m^2^ and obese as BMI >30 kg/m^2^. Abdominal subcutaneous and visceral fat area fractions were obtained from MRI segmentation as described below.

Systolic and diastolic blood pressure (BP) was measured with a digital blood pressure monitor (Model UA-631; A&D Medical, Tokyo, Japan) whilst participants were comfortably seated with their arm supported on a pillow. The average of three BP readings was taken and hypertension was defined as systolic BP ≥ 140 mm Hg. Other cardio-vascular risk factors of diabetes mellitus, high levels of blood cholesterol controlled with statin medication, history of smoking and weekly alcohol intake were self-reported by participants in a medical history questionnaire (Metzler-Baddeley et al., 2013). Information about participants' physical activity over the preceding week was collected with the short version of the International Physical Activity Questionnaire (IPAQ) (Craig et al., 2003). The median number of hours of non-sedentary activities including walking, gardening, housework and moderate to vigorous activities were recorded. Participants’ intellectual function was assessed with the National Adult Reading Test (NART) (Nelson, 1991), cognitive impairment was screened for with the Mini Mental State Exam (MMSE) (Folstein et al., 1975) and depression with the PHQ-9 (Kroenke et al., 2001). All participants had an MMSE ≥26. Eight participants scored ≥10 in the PHQ-9 suggesting moderate levels of depression but no participant was severely depressed.

### 2.3 Blood plasma analysis

Venous fasting blood samples were drawn into 9 ml heparin coated plasma tubes after 12 h overnight fasting and were centrifuged for 10 min at 2,000×*g* within 60 min from blood collection. Plasma samples were transferred into 0.5 ml polypropylene microtubes and stored in a freezer at −80 °C.

Circulating levels of high-sensitivity CRP in mg/dL were assayed using a human CRP Quantikine enzyme-linked immunosorbent assay (ELISA) kit (R & D Systems) and IL-8 levels in pg/mL were determined using a high sensitivity CXCL8/INTERLEUKIN-8 Quantikine ELISA kit (R & D Systems). Leptin concentrations in pg/ml were determined with the DRP300 Quantikine ELISA kit (R & D Systems) and adiponectin in ng/ml with the human total adiponectin/Acrp30 Quantitkine ELISA kit (R & D Systems) and leptin/adiponectin ratios for each participant were calculated (Lopez-Jaramillo et al., 2014). Determination of interleukin-1β, interleukin-6 and Tumor Necrosis Factor α (TNFα) levels were trialed with Quantikine ELISA kits but did not lead to reliable measurements consistently above the level of detection for each assay. All ELISA analyses were carried out in the laboratory of the School of Pharmacy and Pharmaceutical Sciences at Cardiff University.

### 2.4 APOE genotyping

Participants provided a saliva sample using the self-collection kit “Oragene-DNA (OG-500) (Genotek) for DNA extraction and APOE genotyping. APOE genotypes ε2, ε3 and ε4 were determined by TaqMan genotyping of single nucleotide polymorphism (SNP) rs7412 and KASP genotyping of SNP rs429358. Genotyping was successful in a total of 207 participants including 165 out of the 166 individuals that had undergone an MRI scan. The genotypic distribution of those successfully genotyped was reported in Metzler-Baddeley et al., 2019. In addition, participants gave information about their family history (FH) of dementia, i.e. whether a first-grade relative (parent or sibling) was affected by LOAD, vascular dementia or Lewy body disease with dementia.

### 2.5 MRI data acquisition

MRI data were acquired on a 3T MAGNETOM Prisma clinical scanner (Siemens Healthcare, Erlangen, Germany) equipped with a 32-channels receive-only head coil at CUBRIC.

#### 2.5.1 Anatomical MRI

T_1_-weighted anatomical images were acquired with a three-dimension (3D) magnetization-prepared rapid gradient-echo (MP-RAGE) sequence with the following parameters: 256× 256 acquisition matrix, TR = 2300 ms, TE = 3.06 ms, TI = 850 ms, flip angle θ = 9°, 176 slices, 1 mm slice thickness, FOV = 256 mm and acquisition time of ∼6 min.

#### 2.5.2 High angular resolution diffusion imaging (HARDI)

Diffusion data (2 × 2 × 2 mm voxel) were collected with a spin-echo echo-planar dual shell HARDI (Tuch et al., 2002) sequence with diffusion encoded along 90 isotropically distributed orientations (Jones et al., 1999) (30 directions at b-value = 1200 s/mm^2^ and 60 directions at b-value = 2400 s/mm^2^) and six non-diffusion weighted scans with dynamic field correction and the following parameters: TR = 9400 ms, TE = 67 ms, 80 slices, 2 mm slice thickness, FOV = 256 × 256 × 160 mm, GRAPPA acceleration factor = 2 and acquisition time of ∼15 min.

#### 2.5.3 Quantitative magnetization transfer weighted imaging (qMT)

An optimized 3D MT-weighted gradient-recalled-echo sequence (Cercignani and Alexander, 2006) was used to obtain magnetization transfer-weighted data with the following parameters: TR = 32 ms, TE = 2.46 ms; Gaussian MT pulses, duration t = 12.8 ms; FA = 5°; FOV = 24 cm, 2.5 × 2.5 × 2.5 mm^3^ resolution. The following off-resonance irradiation frequencies (Θ) and their corresponding saturation pulse nominal flip angles (ΔSAT) for the 11 MT-weighted images were optimized using Cramer-Rao lower bound optimization: Θ = [1000 Hz, 1000 Hz, 2750 Hz, 2768 Hz, 2790 Hz, 2890 Hz, 1000 Hz, 1000 Hz, 12060 Hz, 47180 Hz, 56360 Hz] and their corresponding ΔSAT values = [332°, 333°, 628°, 628°, 628°, 628°, 628°, 628°, 628°, 628°, 332°]. The longitudinal relaxation time, T_1_, of the system was estimated by acquiring three 3D gradient recalled echo sequence (GRE) volume**s** with three different flip angles (θ = 3°,7°,15°) using the same acquisition parameters as used in the MT-weighted sequence (TR = 32 ms, TE = 2.46 ms, FOV = 24 cm, 2.5 × 2.5 × 2.5 mm^3^ resolution). Data for computing the static magnetic field (B_0_) were collected using two 3D GRE volumes with different echo-times (TE = 4.92 ms and 7.38 ms respectively; TR = 330 ms; FOV = 240 mm; slice thickness 2.5 mm) (Jezzard and Balaban, 1995).

#### 2.5.4 Abdominal scans

The Dixon technique (Dixon, 1984) was used to estimate the fat content of the abdominen at the level of the lumbar vertebral body 4. A pair of single slice 2D TurboFLASH images (fast gradient echo with inversion recovery; TR_1_ = 1910 ms, TR_2_ = 5.9 ms, TI = 1200 ms, flip angle θ = 20°, Field of View = 480 × 390 mm; acquisition matrix = 256 × 166; 10 mm slice thickness, 3.4 s acquisition time) was acquired. The echo times were set such that one image was acquired with water and fat signals in-phase (TE = 2.34 ms), and one with them out of phase (TE = 3.4 msec). Participants were instructed to hold their breath during the brief image acquisition to minimise movement artefacts.

### 2.6 MRI data processing

The two-shell diffusion-weighted HARDI data were split and b = 1200 and 2400 s/mm^2^ data were corrected separately for distortions induced by the diffusion-weighted gradients and artifacts due to head motion with appropriate reorientation of the encoding vectors (Leemans and Jones, 2009) in ExploreDTI (Version 4.8.3) (Leemans et al., 2009). EPI-induced geometrical distortions were corrected by warping the diffusion-weighted image volumes to the T_1_ –weighted anatomical images, which were down-sampled to a resolution of 1.5 × 1.5 × 1.5 mm (Irfanoglu et al., 2012). After preprocessing, the Neurite Orientation Dispersion and Density (NODDI) model (Zhang et al., 2012) was fitted to the dual-shell HARDI data using fast, linear model fitting algorithms of the Accelerated Microstructure Imaging via Convex Optimization (AMICO) framework (Daducci et al., 2015) to obtain ISOSF, ICSF and ODI maps (Fig. 1).

MT-weighted GRE volumes for each participant were co-registered to the MT-volume with the most contrast using a rigid body (6 degrees of freedom) registration to correct for inter-scan motion using Elastix (Klein et al., 2010). Data from the 11 MT-weighted GRE images and T1-maps were fitted by a two-pool model using the pulsed-MT approximation proposed by Ramani et al. (2002). This approximation provided maps of MPF and the forward exchange rate *k*_*f*_. MPF maps were thresholded to an upper intensity limit of 0.3 and *k*_*f*_ maps to an upper limit of 3 using the FMRIB's fslmaths imaging calculator to remove voxels with noise-only data.

All image modality maps and region of interest masks were spatially aligned to the T_1_-weighted anatomical volume as reference image with linear affine registration (12 degrees of freedom) using FMRIB's Linear Image Registration Tool (FLIRT).

### 2.7 Tractography

The RESDORE algorithm (Parker, 2014) was applied to identify outliers, followed by whole brain tractography with the damped Richardson-Lucy algorithm (dRL) (Dell'acqua et al., 2010) on the 60 direction, b = 2400 s/mm^2^ HARDI data for each dataset in single-subject native space using in house software (Parker, 2014) coded in MATLAB (the MathWorks, Natick, MA). To reconstruct fibre tracts, dRL fibre orientation density functions (fODFs) were estimated at the centre of each image voxel. Seed points were positioned at the vertices of a 2 × 2x2 mm grid superimposed over the image. The tracking algorithm interpolated local fODF estimates at each seed point and then propagated 0.5 mm along orientations of each fODF lobe above a threshold on peak amplitude of 0.05. Individual streamlines were subsequently propagated by interpolating the fODF at their new location and propagating 0.5 mm along the minimally subtending fODF peak. This process was repeated until the minimally subtending peak magnitude fell below 0.05 or the change of direction between successive 0.5 mm steps exceeded an angle of 45°. Tracking was then repeated in the opposite direction from the initial seed point. Streamlines whose lengths were outside a range of 10 mm–500 mm were discarded.

The fornix, PHC, UF and CST pathways were reconstructed with an in-house automated segmentation method based on PCA of streamline shape (Parker et al., 2012). This procedure involves the manual reconstruction of a set of tracts that are then used to train a PCA model of candidate streamline shape and location. Twenty datasets were randomly selected as training data. Tracts were reconstructed by manually applying waypoint region of interest (ROI) gates (“AND”, “OR” and “NOT” gates following Boolean logic) to isolate specific tracts from the whole brain tractography data. ROIs were placed in HARDI data native space on colour-coded fiber orientation maps (Pajevic & Pierpaoli, 1999) in ExploreDTI following published protocols as reported in Metzler-Baddeley et al (2011, 2012a, 2012b, 2013). The trained PCA shape models were then applied to all datasets: candidate streamlines were selected from the whole volume tractography as those bridging the gap between estimated end points of the candidate tracts. Spurious streamlines were excluded by means of a shape comparison with the trained PCA model. All automatic tract reconstructions underwent quality control through visual inspection and any remaining spurious fibers that were not consistent with the tract anatomy were removed from the reconstruction where necessary. Mean values of all qMT and NODDI indices (Fig. 1, Table 2) were extracted for all white matter pathways.

### 2.8 Whole brain white matter and hippocampal segmentation

Whole brain white matter, left and right whole hippocampus masks were automatically segmented from T_1_-weighted images with the Freesurfer image analysis suite (version 5.3), which is documented online (https://surfer.nmr.mgh.harvard.edu/). The hippocampal regions included areas of the presubiculum, subiculum, cornu ammonis subfields 1–4, dentate gyrus, hippocampal tail and fissure but excluded cortical regions such as the entorhinal cortex (Iglesias et al., 2015, 2016). Whole brain white matter masks were thresholded to exclude ventricle cerebrospinal fluid spaces from the mask. Mean values of all qMT and NODDI indices (Fig. 1, Table 2) were extracted for the whole brain white matter mask and the left and right hippocampus masks.

### 2.9 Abdominal subcutaneous and visceral fat area segmentation

All images were visually inspected for motion artefacts and in- and out-phase alignment in MRIcron (Rorden et al., 2007). Pure fat signal images were created with the fslmaths tool from the FSL analysis library (Jenkinson et al., 2012; Smith et al., 2004) by subtracting out-phase images of the water signal from in-phase images that contained signals from both fat and water (Dixon, 1984). Subcutaneous and visceral fat regions were then manually segmented from fat-only images in fslview. Subcutaneous fat was defined as fat tissue exterior to the abdominal wall (see Fig. 1c). Visceral fat regions were isolated by removing subcutaneous fat, muscle tissue (left and right psoas muscles; left and right internal and external oblique muscles; left and right transversus abdominis muscles; left and right rectus abdominus muscles), and the spinal disc from the images (Fig. 1c). Subcutaneous and visceral masks were then binarized and individually thresholded to ensure that only fat tissue was included in the masks. Finally, subcutaneous and visceral fat area fractions were obtained by dividing subcutaneous and visceral fat area by the total abdominal fat area.

### 2.10 Statistical analyses

Statistical analyses were conducted in SPSS version 24 (IBM, 2011) and the PROCESS computational tool for mediation analysis (Hayes, 2012). All data were inspected for assumptions of normal distribution and variance heterogeneity. Plasma adipokines, CRP and IL-8 were log-transformed to correct for skew. All multiple-comparisons-related Type 1 errors were corrected with a 5% False Discovery Rate (FDR) using Benjamini-Hochberg adjusted p-values. All p-values were two-tailed. Partial Eta^2^ (ηp^2^) and correlation coefficients are reported as indices of effect sizes.

Omnibus multivariate regression analysis was conducted to test for the relationships between the body composition/adiposity metrics and demographic variables (sex, age, years of education), health-related variables (alcohol consumption, blood pressure, physical activity, plasma leptin/adiponectin ratio, CRP, and IL-8) and genetic risk of LOAD (*APOE* genotype, FH). Post-hoc group comparisons were conducted with independent t-tests and were 5% FDR corrected.

PCA was employed for the purpose of assessing data dimensionality and of reducing data complexity. This was done separately for white and grey matter as the two tissue types differ in their microstructural organisation, i.e., white matter consists of relatively aligned axons and glia, whilst grey matter comprises a more complex microstructure of cell bodies, dendrites, synapses and glia. PCA was also employed to reduce the complexity of the microstructural data [40 measurements for white matter (5 MRI indices x 8 regions of interest, Table 3) and 10 measurements for the left and right hippocampi (Table 4)] for subsequent correlation analyses with the body composition/adiposity metrics. A PCA procedure with orthogonal Varimax rotation of the component matrix that used the Kaiser criterion of including all components with an eigenvalue of >1(IBM, 2011) was used. Cattell's scree plot (Cattell, 1952) and component loadings were inspected with regard to their interpretability. Loadings that exceed a value of 0.5 were considered as “significant”.

**Table 4:** Rotated component matrix of the principal component analysis of the hippocampal microstructural indices.^a^.

*ROI*	*GM index*	Components			
		ODI/MPF	*ISOSF*	ICSF	*k*_*f*_
Left HC	ICSF	0.047	−0.058	**0.941**	0.066
	ISOSF	0.104	**0.877**	−0.015	−0.174
	ODI	**0.834**	0.106	0.005	−0.246
	MPF	**−0.537**	−0.378	0.236	−0.195
	k_f_	−0.044	−0.118	−0.02	**0.859**
Right HC	ICSF	−0.191	−0.078	**0.901**	−0.033
	ISOSF	0.153	**0.846**	−0.077	−0.262
	ODI	**0.863**	0.025	0.005	−0.217
	MPF	**−0.526**	−0.437	0.218	−0.115
	k_f_	−0.194	−0.189	0.05	**0.807**

Loadings >0.5 are highlighted in bold. Abbreviations: GM = grey matter, HC = hippocampus, ICSF = intracellular signal fraction, ISOSF = isotropic signal fraction, *kf* = forward exchange rate, MPF = macromolecular proton fraction, ODI = orientation dispersion index, ROI = region of interest.

a Rotation method: Varimax with Kaiser normalization.

Pearson correlation coefficients were then calculated between body composition/adiposity metrics, and white and grey matter microstructural components, as well as those fornix microstructural indices that loaded highly on the fornix component (Table 3). These correlations were 5% FDR corrected. Correlations were then further explored for the contribution of any confounding variables identified in the multivariate omnibus regression analysis (i.e. sex, age, leptin/adiponectin ratio, CRP). The obesity-brain correlations controlling for age in the whole sample and the correlations that were carried out separately for men and women were 5% FDR corrected.

**Table 3:** Rotated component matrix of the principal component analysis of the white matter microstructural indices^a^.

*ROI*	*WM index*	Components				
		ISOSF/ODI	*k*_*f*_	MPF	ICSF	Fornix
Fornix	ICSF	−0.201	−0.039	−0.081	0.497	0.431
	ISOSF	−0.045	0.012	−0.015	0.039	**−0.93**
	ODI	**0.622**	−0.041	−0.027	−0.122	−0.364
	MPF	−0.114	0.041	0.458	0.049	**0.782**
	k_f_	−0.09	**0.525**	−0.014	0.008	**0.757**
Left PHC	ICSF	0.264	0.107	0.101	**0.792**	−0.191
	ISOSF	**0.781**	−0.045	0.01	0.11	−0.042
	ODI	**0.641**	−0.251	−0.091	−0.287	0.123
	MPF	0.124	0.276	**0.552**	0.347	0.005
	k_f_	0.09	**0.814**	−0.048	0.18	−0.004
Right PHC	ICSF	0.116	0.054	0.225	**0.77**	−0.233
	ISOSF	**0.752**	−0.008	0.09	−0.08	−0.081
	ODI	**0.621**	−0.086	−0.155	−0.285	0.206
	MPF	0.049	0.149	**0.701**	0.265	−0.01
	k_f_	0.007	**0.82**	0.026	0.156	0.107
Left UF	ICSF	−0.043	0.075	0.377	**0.743**	0.007
	ISOSF	**0.728**	−0.062	0.234	−0.083	−0.029
	ODI	**0.831**	−0.054	−0.066	−0.103	0.028
	MPF	0.018	0.262	**0.76**	0.182	0.04
	k_f_	−0.154	**0.814**	0.178	0.108	−0.054
Right UF	ICSF	−0.039	0.09	0.427	**0.647**	0.089
	ISOSF	**0.642**	−0.021	0.226	−0.28	0.066
	ODI	**0.781**	−0.03	−0.025	−0.154	0.039
	MPF	−0.027	0.204	**0.772**	0.086	0.002
	k_f_	0.04	**0.752**	0.351	0.005	−0.043
Left CST	ICSF	−0.222	0.02	0.031	**0.66**	0.085
	ISOSF	**0.742**	−0.087	−0.032	0.077	−0.088
	ODI	**0.797**	0.029	0.022	0.036	0.097
	MPF	−0.067	−0.045	**0.83**	0.132	0.042
	k_f_	−0.155	**0.818**	0.107	−0.088	0.073
Right CST	ICSF	−0.271	0.049	0.147	**0.56**	0.079
	ISOSF	**0.716**	−0.116	−0.127	0.115	−0.132
	ODI	**0.826**	0.026	−0.053	0.064	0.065
	MPF	−0.114	−0.024	**0.85**	0.077	−0.015
	k_f_	−0.167	**0.802**	0.169	−0.067	0.069
Total WM	ICSF	−0.298	0.001	0.267	**0.701**	−0.333
	ISOSF	**0.752**	−0.037	−0.105	−0.147	0.201
	ODI	**0.527**	0.06	−0.186	0.005	−0.195
	MPF	−0.065	0.1	**0.812**	0.196	0.235
	k_f_	−0.095	**0.928**	0.051	0.047	0.062

Loadings >0.5 are highlighted in bold. Abbreviations: CST = corticospinal tract, ICSF = intracellular signal fraction, ISOSF = isotropic signal fraction, *kf* = forward exchange rate, MPF = macromolecular proton fraction, ODI = orientation dispersion index, PHC = parahippocampal cingulum, ROI = region of interest, UF = uncinate fasciculus, WM = white matter.

a Rotation method: Varimax with Kaiser normalization.

Finally, linear mediation analysis was employed to test for the indirect effects (Fig. 3D–F, path a*b) of inflammation mediator variables on the direct effects (Fig. 3D–F, path c’) of obesity on brain microstructure. The significance of indirect and direct effects was assessed with a 95% confidence interval based on bootstrapping with 5000 replacements (Hayes, 2012).

### 2.11 Missing data

Four participants did not complete the 90 min MRI scanning session due to claustrophobia and qMT and abdominal MRI data are missing for these individuals. The abdominal scans were acquired at the end of the MR session and thirty-two abdominal datasets had to be excluded from the analyses due motion artefacts and due to participants holding their breath at different points of the breathing cycle during in and out-phase image acquisition, i.e. at the end of an exhalation in one image and at the end of an inhalation in the other. This meant that the image pairs were not spatially aligned and hence subcutaneous and visceral fat regions could not be reliably delineated. Furthermore, bloods could not be drawn or analysed for 18 participants. For one participant *APOE* could not be genotyped from the saliva sample and two participants did not know their family history of dementia.

## 3 Results

### 3.1 Sex-related differences in demographic, health and genetic variables

Men and women were comparable with regards to their age, years of education, their performance in the NART and MMSE, their genetic risk of dementia, their weekly physical activities, and the number of smokers and diabetics (Table 1). However, men were more centrally obese (WHR) with higher VFF but lower SFF than women (Table 1). Men reported larger alcohol consumption than women, and more men than women were on statin medication (Table 1). Leptin and adiponectin levels were higher in women than men, but CRP and IL-8 levels did not differ between the two groups (Table 1).

### 3.2 Cross-correlations between body composition/adiposity metrics

Individual differences in BMI correlated positively with differences in the SFF [r(130) = 0.4, p < 0.001], and with differences in WHR [r(166) = 0.21, P = 0.006] but not with differences in VFF (p = 0.29). Differences in WHR correlated positively with differences in VFF [r(130) = 0.28, p = 0.001] but not with SFF (p = 0.7). There was a negative correlation between differences in VFF and SFF [r(130) = −0.42, p < 0.001].

### 3.3 Multivariate regression analysis exploring the relationship between body composition/adiposity metrics and demographic, health and genetic variables

Multivariate regression analysis was employed to test for any potential effects of demographic variables (age, sex, years of education), health-related variables (alcohol consumption, systolic and diastolic blood pressure, physical activity, plasma leptin-adiponectin ratio, CRP, IL-8), and genetic risk of LOAD (*APOE* genotype, FH) on the body composition/adiposity metrics (BMI, WHR, SFF and VFF).

There were significant omnibus effects of sex [F(4, 85) = 13.9, p < 0.001, ηp^2^ = 0.4], age [F(4, 85) = 2.8, p = 0.03; ηp^2^ = 0.12], leptin-adiponectin ratio [F(4, 85) = 9.9, p < 0.001, ηp^2^ = 0.32] and CRP [F(4, 85) = 2.8, p = 0.03, ηp^2^ = 0.12] on the four adiposity metrics.

Omnibus effects were followed up by 5% FDR corrected post-hoc comparisons. Men had higher WHR [t(164) = 5.5, p < 0.001] and VFF [t(128) = 3.1, p = 0.002] than women (Table 1). Older individuals over 60 had larger VFF than younger individuals in their 40 ies [t(82) = 3.24, p = 0.002]. The leptin-adiponectin ratio was positively correlated with BMI [r(148) = 0.59, p < 0.001] and SFF [r(115) = 0.46, p < 0.001], and CRP correlated positively with BMI [r(145) = 0.51, p < 0.001] and WHR [r(145) = 0.23 p = 0.005]. There was also a positive correlation between the leptin-adiponectin ratio and CRP [r(145) = 0.47, p < 0.001].

### 3.4 Dimensionality of microstructural indices in white matter and hippocampal grey matter

Table 3 summarizes the component loadings for the extracted WM components. PCA resulted in five components that all exceeded an eigenvalue of 2 and explained together 65.4% of the data variation. The first component had high loadings on ISOSF and ODI, the second on *k*_*f*_, the third on MPF, and the fourth on ICSF in all white matter regions. The fifth component had loadings from ISOSF, MPF and *k*_*f*,_ of the fornix only.

PCA of microstructural metrics from left and right hippocampi extracted four components that accounted for 75% of the data. These components were: 1st ODI and MPF, 2nd ISOSF, 3rd ICSF, and 4th *k*_f_ from left and right hippocampi (Table 4).

### 3.5 Correlations between body composition/adiposity metrics and white and grey matter microstructure

**Fig. 2:**
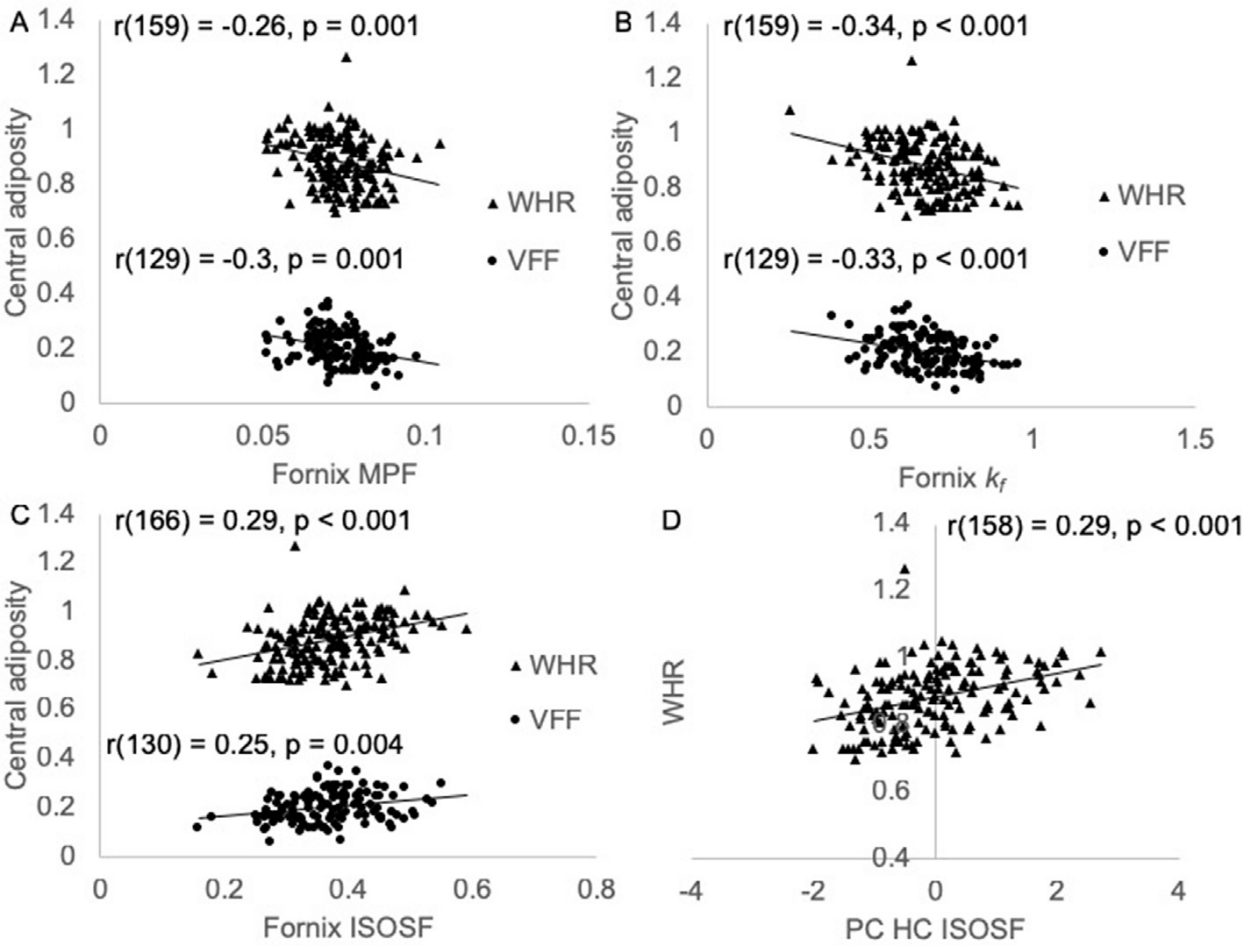
Plots the following obesity-brain correlations for the whole group of participants. A) Fornix macromolecular proton fraction (MPF) was negatively correlated with Waist-to-Hip Ration (WHR) and visceral area fraction (VFF). B) Fornix forward exchange rate *k*_*f*_ was negatively associated with WHR and VFF. C) Fornix isotropic signal fraction (ISOSF) was positively correlated with WHR and VFF. D) Positive correlations between WHR and the hippocampal ISOSF component. All p-values were 5% false discovery rate (FDR) corrected. Note that three extreme outliers in the WHR variable, that deviated more than three standard deviations from the regression slope, were removed from the scatterplot for display purposes, but their removal did not alter the results.

Pearson correlation coefficients were calculated between BMI, WHR, SFF, and VFF and the following brain components: WM ODI/ISOSF, WM ICSF, WM MPF, WM *k*_*f*_, hippocampal ODI/MPF, hippocampal ISOSF, hippocampal ICSF and hippocampal *k*_*f*_ (Table 3, Table 4). As fornix was a separate component in the PCA with high loadings from fornix MPF, *k*_*f*_, and ISOSF (Table 3) these variables were also included in the analyses. The following Pearson correlation coefficients were significant after 5% FDR correction. WHR was negatively associated with fornix MPF [r(162) = −0.3, p < 0.001] (Fig. 2A) and fornix *k*_*f*_ [r(162) = −0.28, p < 0.001] (Fig. 2B) and positively associated with fornix ISOSF [r(166) = 0.29, p < 0.001] (Fig. 2C) and the hippocampal ISOSF component [r(158) = 0.29, p < 0.001] (Fig. 2D). VFF correlated negatively with fornix MPF [r(129) = −0.3, p = 0.001] (Fig. 2A) and fornix *k*_*f*_ [r(129) = −0.33, p < 0.001] (Fig. 2B) and positively with fornix ISOSF [r(130) = 0.25, p = 0.004] (Fig. 2C).

### 3.6 Effects of age and sex on obesity-brain correlations

**Fig. 3:**
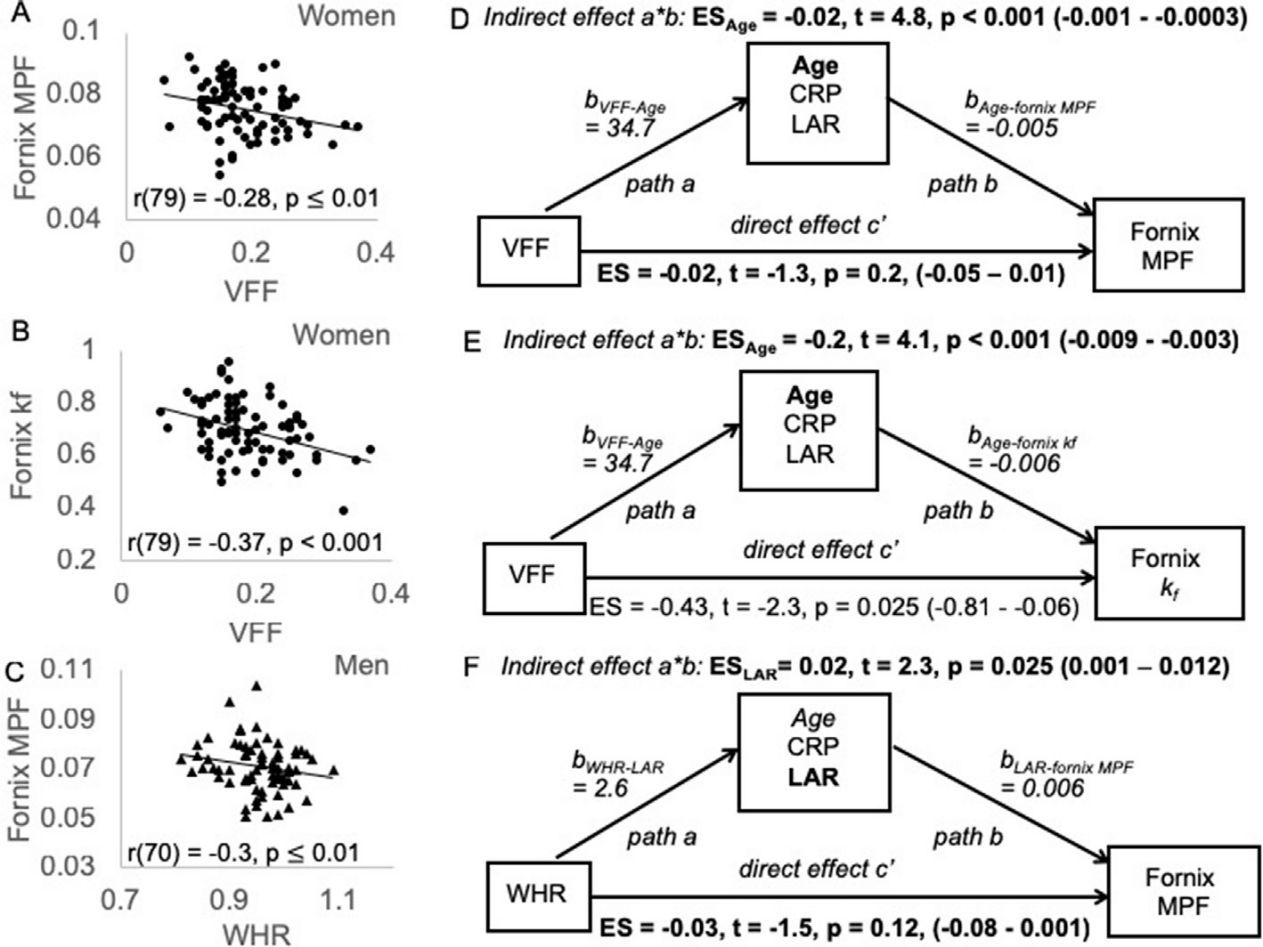
Women showed negative correlations between differences in visceral fat area fraction (VFF) and differences in the fornix macromolecular proton fraction (MPF) (A) and *k*_*f*_ (B). C) Men showed a negative correlation between differences in waist hip ratio (WHR) and fornix MPF. D) For women, mediation analysis revealed that age fully mediated the correlation between VFF and fornix MPF (highlighted in bold) and also contributed to the correlation between VFF and fornix *k*_*f*_ (E). For men differences in the leptin/adiponectin ratio (LAR) fully mediated the correlation between WHR and fornix MPF. There was also a trend for a mediating effect of age (p = 0.05). 95% confidence interval in brackets were based on bootstrapping with 5000 replacements. ES = Effect size, b = unstandardized coefficients.

As age and sex had significant effects on WHR and VFF, we assessed their respective effects on the observed adiposity-brain correlations by firstly partialling out age, and secondly, by calculating Pearson correlations coefficients for men and women separately. After 5% FDR correction, the correlations between WHR and fornix MPF [r(123) = −0.24, p = 0.008], fornix *k*_*f*_ [r(123) = −0.24, p = 0.008] and hippocampal ISOSF component [r(123) = −0.3, p = 0.001] remained significant when age was partialled out. Women showed significant negative correlations between VFF and fornix MPF [r(79) = −0.28, p = 0.01] (Fig. 3A) and fornix *k*_*f*_ [r(79) = −0.37, p < 0.001] (Fig. 3B), and men between WHR and fornix MPF [r(70) = −0.3, p = 0.01] (Fig. 3C). No correlations with ISOSF were present separately for men (r_WHR-fornix ISOSF_ = 0.2, p = 0.07; r_WHR-HC ISOSF component_ = 0.15, p = 0.23) or women (r_WHR-fornix ISOSF_ = 0.08, p = 0.47; r_WHR-HC ISOSF component_ = 0.16, p = 0.14).

### 3.7 Mediation analysis exploring the contribution of systemic inflammation

We then carried out mediation analyses to explore the contributing effects of individual differences in CRP, the leptin/adiponectin ratio, and age for men and women separately. In women, age fully mediated the effects of VFF on fornix MPF (Fig. 3D) but not on fornix *k*_*f*_ (Fig. 3E), without any additional contributions of blood immunity measures. In men, differences in the leptin/adiponectin ratio and in age fully removed the direct effect of WHR on fornix MPF (Fig. 3F).

## 4 Discussion

Midlife obesity is a risk factor of LOAD but the biological mechanisms underpinning this link remain poorly understood. Both conditions are associated with systemic inflammation, and it is increasingly recognised that microglia-mediated immune responses play an important role in LOAD (Dansokho and Heneka, 2018; Heneka et al., 2015; Sarlus and Heneka, 2017; Tejera and Heneka, 2016). It has therefore been proposed that obesity induced gut dysbiosis may trigger microglia mediated neuroinflammation, and in turn may contribute to the development of LOAD pathology (Bartzokis, 2011; Sochocka et al., 2018; Venegas et al., 2017). If that was the case, one may expect adverse effects of obesity-related neuroinflammation, such as white matter myelin damage (Serres et al., 2009b), that may occur independently of axonal injury (Bitsch et al., 2000), to manifest in brain regions involved in LOAD. Furthermore, as the pathological processes leading to LOAD are likely to accumulate over many years (Jack et al., 2013), it may be possible to identify such brain tissue changes in asymptomatic individuals prior to the onset of any memory symptoms.

Here, we investigated the impact of systemic inflammation on the relationship between central obesity and brain microstructure in 166 asymptomatic individuals from CARDS that were well characterised with regards to their lifestyle and genetic risk of LOAD (Table 1). Going beyond previously adopted DTI analyses with qMT and NODDI, we dissociated obesity-related effects on myelin/inflammation/metabolism from neurite density/orientation components in white and grey matter limbic regions. Whilst individual differences in central obesity, i.e. WHR and VFF, were negatively correlated with differences in fornix MPF and *k*_*f*_*,* no effects were observed for NODDI ICSF or ODI, suggesting that central obesity impairs apparent myelin/inflammatory properties rather than apparent axon loss in white matter. Further analyses revealed that in women, age mediated the negative correlations between VFF and fornix MPF and *k*_*f*._ This was due to age-related increases in VFF and reductions in fornix MPF and *k*_*f*_ (Metzler-Baddeley et al., 2019). In men, however, individual differences in the leptin/adiponectin ratio (with a trend for age) fully mediated the negative correlation between WHR and fornix MPF. As men were significantly more centrally obese due to larger VFF than women, these results suggest that obesity-related changes in inflammatory states may contribute to apparent myelin damage and neuroinflammation in the fornix. Excessive VFF is known to be associated with a shift in the ratio between leptin and adiponectin, due to increases in leptin and reductions in adiponectin (Lopez-Jaramillo et al., 2014), resulting in inflammatory states that may exacerbate neuroinflammation (Ouchi and Walsh, 2012) and associated white matter myelin damage (Bartzokis, 2011).

Whilst reductions in MPF may reflect obesity related damage of axon myelin sheaths, it may also be possible that obesity increases the number of smaller axons with fewer lamellae in the myelin sheath. However, we did not observe any effects on axon density and orientation indices from NODDI, which might be expected if the latter was the case.

Men reported higher alcohol consumption, that may have contributed to their increased VFF. However, differences in the number of weekly consumed alcohol units did not correlate with the leptin/adiponectin ratio in men (r = 0.16, p = 0.2), and alcohol had no overall effect on adiposity metrics in the multivariate analysis. Thus, we would argue that shifts in the leptin/adiponectin ratio in men were primarily due to their central obesity.

In contrast to the obesity effects on fornix MPF and *k*_*f*_, the apparent correlations with fornix and hippocampal ISOSF metrics were solely driven by sex differences in central obesity and ISOSF measures, i.e. men showed higher fornix and hippocampal ISOSF than women (Metzler-Baddeley et al., 2019).

Correlation analyses revealed positive correlations between WHR and VFF but not with SFF. In contrast, BMI correlated positively with SFF but not with VFF. Whilst BMI and WHR were positively correlated, VFF and SFF correlated negatively with each other. From this pattern of cross-correlations, it is clear that BMI and WHR captured quite different fat distributions in our sample. Indeed, negative correlations with white matter microstructure were only observed for WHR and VFF, but not for BMI or SFF. These results are consistent with previous findings of visceral but not subcutaneous fat being associated with an increased risk of metabolic syndrome and mortality (Koster et al., 2010, 2015; Koster and Schaap, 2015), as well as with reduced brain volume (Debette et al., 2010).

The observed pattern of correlations also implies that the direct comparison of results across studies with different measures of body obesity/distribution may be difficult. This observation may explain discrepant findings in the literature, as some studies reported beneficial effects of larger BMI or waist circumference on white matter microstructure (Birdsill et al., 2017), whilst other studies reported adverse effects (Kullmann et al., 2015; Ronan et al., 2016). Previously we reported significant correlations between BMI and fornix white matter microstructure in a smaller group of older adults (Metzler-Baddeley et al., 2013), a result that was not replicated here. At first glance these findings seem at odds. However, the first study investigated white matter microstructure with DTI indices of fractional anisotropy (FA), mean, axial, and radial diffusivity, and observed positive correlations between BMI and the diffusivities, but no correlation with FA. DTI indices are non-specific metrics of white matter microstructure that are affected by changes in biological white matter properties as well as by their geometrical and organisational architecture (Beaulieu and Allen, 1994; De Santis et al., 2014). Hence it is not possible to interpret BMI-related increases in fornix diffusivities in terms of differences in myelin or axon density. Furthermore, participants in the first study were older adults with BMI levels from within the normal to overweight range (53–93 years of age, Mean_age_ = 68, Mean_BMI_ = 24.9, n = 38), whilst the majority of middle-aged participants in this study were overweight (Mean_BMI_ = 27) with 20% falling within the obese category. As we proposed in the previous paper, the observed relationship between BMI and fornix microstructure may not relate to mechanisms underpinning obesity but may rather reflect some functional properties of the fornix within hippocampal-hypothalamic-prefrontal food control networks (Metzler-Baddeley et al., 2013).

The results of the PCA for the white matter microstructural indices were consistent with our assumption that MPF, *k*_*f*_ and ICSF provide estimates of different white matter tissue properties, i.e. of apparent myelin, inflammation-related tissue metabolism, and apparent axon density. ODI and ISOSF, however, were jointly loading on one component, suggesting that in our dataset they captured overlapping microstructural features. Exploratory PCA of the microstructural indices in hippocampal grey matter revealed separate components for *k*_*f*_, ICSF, and ISOSF but here ODI and MPF were found to load jointly on one component. This suggests that qMT and NODDI indices may not be directly comparable across white and grey matter. As they have primarily been validated in white matter (Sled, 2017; Zhang et al., 2012), their interpretation in grey matter remains speculative and requires histological validation.

The correlations between WHR and VFF and myelin/inflammation-sensitive metrics were specifically observed in the fornix tract but not for microstructural components across the other white matter regions of interest. As the fornix is known to be impaired in Mild Cognitive Impairment and early LOAD (Oishi and Lyketsos, 2014; Oishi et al., 2012; Plowey and Ziskin, 2016; Yu et al., 2017), this pattern of results is consistent with the view that visceral fat may be associated with processes that have adverse effects on limbic areas involved in LOAD. Indeed, we recently reported for the same CARDS participants, that age-related reductions in fornix MPF and *k*_*f*_ fully mediated age-related decline in hippocampal tissue but not *vice versa*, suggesting that fornix glia-related damage due to ageing and obesity, may cause hippocampal damage (Metzler-Baddeley et al., 2019). Furthermore, animal studies have shown that diet-induced obesity can trigger microglia-mediated inflammation in the hippocampus that impairs synaptic functioning and spatial memory (Hao et al., 2016).

The relationship between obesity and genetic risk of dementia, notably *APOE* genotype, remains not fully understood. Whilst some evidence suggests that obesity may modulate the association between *APOE* genotype and fasting insulin and glucose levels, and hence potentially inflammation in men (Elosua et al., 2003), other studies point to independent effects of obesity, diabetes, and *APOE* genotype to LOAD risk (Profenno et al., 2010). Here we did not observe any effects of *APOE* genotype or of family history of dementia on obesity status for the whole group, and *APOE* genotype did not have any mediating effects on the observed WHR-fornix MPF correlation in men (p = 0.68). However, we did not specifically investigate insulin resistance and glucose metabolism in the present study, and our sample size may not have been sufficiently large to detect any interactive effects between APOE genotype and obesity-related mechanisms in men.

In our study we also did not observe specific effects on hippocampal microstructure. However, for the reasons outlined above, one may expect qMT metrics to be more sensitive to myelin damage in white rather than in grey matter, especially as we were studying a sample of asymptomatic individuals. Our results are also consistent with a previous study that used multi-parameter mapping and reported BMI-related differences in MRI metrics consistent with reduced myelin, increased water and changes in tissue iron content in tracts connecting limbic structures with the prefrontal cortex (Kullmann et al., 2016).

A few methodological limitations of our study need to be recognised. Firstly, although the Ramani 'Continuous Wave Power Equivalent' approximation for pulsed MT experiments reduces the complexity in modelling the data, it does introduce a bias into the fitted parameters. Most relevant for the data presented here, it can lead to a consistent underestimation in the exchange rate, *k*_*f*_. However, the Ramani approximation provides a robust estimate of MPF, as noted by Portnoy and Stanisz (2007).

Secondly, we were not able to acquire maps of the B1 transmit field as part of the study. Variations in the B1 transmit field generated by the RF coil will introduce a spatial variation in the effective MT pulse amplitude. Thus, the effective MT weighting for each MT acquisition will vary across the brain. This can be corrected by measuring this B1 transmit inhomogeneity by using, for example, the Bloch-Siegert method (Sacolick et al., 2010). It has been reported that B1 field errors will result in variations in the fitted value of the MPF (Sled and Pike, 2001), however we do not believe that this would explain the findings in this work.

Although we observed an omnibus effect of CRP on all body adiposity measures consistent with previous evidence (Verstynen et al., 2013), differences in CRP did not mediate the central obesity-fornix myelin/neuroinflammation correlations and differences in IL-8 had no overall effect. These results should be seen within the context that in our cohort of middle-aged healthy participants without clinical inflammatory conditions, plasma concentrations of inflammatory markers such as interleukin-1β, interleukin-6 and Tumor Necrosis Factor α were generally below the limit of detection of the assays, and we had to employ high sensitivity ELISA kits to measure CRP and IL-8. Thus, we cannot rule out that in our study the effects of systemic inflammation on obesity-brain relationships were underestimated due to relatively low levels of inflammatory markers in our cohort of healthy individuals.

To summarise, the present study showed that central obesity, notably abdominal visceral fat accumulation, was associated with differences in qMT indices of apparent myelin and neuroinflammation in the fornix in a sample of asymptomatic individuals at midlife and early older age. In men, who had larger VFF than women, these correlations were mediated by shifts in the leptin/adiponection ratio, suggesting that visceral fat related inflammatory states contribute to myelin-related damage in the fornix. As the fornix is a key limbic structure involved in LOAD, these results are consistent with the view that obesity-related changes in immune responses and associated white matter glia changes may contribute to the link between midlife obesity and LOAD.

## 5 Author contributions

CMB is the PI of the study and is responsible for the conceptualisation and data acquisition and analyses of the study. CMB has also written the manuscript. JPM and EL were responsible for participant recruitment, data acquisition and MRI data processing. RS was responsible for the *APOE* genotyping. FF and JE have prepared the qMT and diffusion MRI protocols and have helped with MRI data processing. AKM and FG carried out the manual segmentations of the abdominal fat area regions. RJB was involved in the conceptualisation and has advised on statistical data analysis. EK and BE were responsible for the ELISA serum analyses. DKJ provided feedback on the study design and manuscript.
